# A sex-specific propensity-adjusted analysis of colonic adenoma detection rates in a screening cohort

**DOI:** 10.1038/s41598-021-97163-0

**Published:** 2021-09-07

**Authors:** Sarah Wernly, Bernhard Wernly, Georg Semmler, Sebastian Bachmayer, David Niederseer, Felix Stickel, Ursula Huber-Schönauer, Elmar Aigner, Christian Datz

**Affiliations:** 1 Department of Internal Medicine, General Hospital Oberndorf, Teaching Hospital of the Paracelsus Medical University Salzburg, Paracelsusstraße 37, Oberndorf, 5110 Salzburg, Austria; 2 Clinic of Internal Medicine II, Department of Cardiology, Paracelsus Medical University of Salzburg, Salzburg, Austria; 3 Department of Medicine, Karolinska Institutet, Karolinska University Hospital, Stockholm, Sweden; 4 Department of Internal Medicine III, Division of Gastroenterology and Hepatology, Medical University of Vienna, Vienna, Austria; 5 Department of Cardiology, University Hospital Zurich, Zurich, Switzerland; 6 Department of Gastroenterology and Hepatology, University Hospital of Zurich, Zürich, Switzerland; 7 First Department of Medicine, Paracelsus Medical University Salzburg, Salzburg, Austria

**Keywords:** Cancer epidemiology, Gastrointestinal cancer, Gastroenterology, Risk factors

## Abstract

The prevalence of colorectal adenoma and advanced adenoma (AA) differs between sexes. Also, the optimal age for the first screening colonoscopy is under debate. We, therefore, performed a sex-specific and age-adjusted comparison of adenoma, AA and advanced neoplasia (AN) rates in a real-world screening cohort. In total, 2824 asymptomatic participants between 45- and 60-years undergoing screening colonoscopy at a single-centre in Austria were evaluated. 46% were females and mean age was 53 ± 4 years. A propensity score for being female was calculated, and adenoma, AA and AN detection rates evaluated using uni- and multivariable logistic regression. Sensitivity analyses for three age groups (group 1: 45 to 49 years, n = 521, 41% females, mean age 47 ± 1 years; group 2: 50 to 54 years, n = 1164, 47% females, mean age 52 ± 1 years; group 3: 55 to 60 years, n = 1139, 46% females, mean age 57 ± 2 years) were performed. The prevalence of any adenoma was lower in females (17% vs. 30%; OR 0.46, 95% CI 0.38–0.55; p < 0.001) and remained so after propensity score adjustment for baseline characteristics and lifestyle factors (aOR 0.52, 95% CI 0.41–0.66; p < 0.001). The same trend was seen for AA with a significantly lower prevalence in females (3% vs. 7%; OR 0.38, 95% CI 0.26–0.55; p < 0.001) that persisted after propensity score adjustment (aOR 0.54, 95% CI 0.34–0.86; p = 0.01). Also, all age-group sensitivity analyses showed lower adenoma, AA and AN rates in females. Similar numbers needed to screen to detect an adenoma, an AA or AN were found in female age group 3 and male age group 1. Colorectal adenoma, AA and AN were consistently lower in females even after propensity score adjustment and in all age-adjusted sensitivity analyses. Our study may add to the discussion of the optimal age for initial screening colonoscopy which may differ between the sexes.

## Introduction

The optimal age recommendations for starting colorectal cancer (CRC) screening are a matter of debate. The American Cancer Society (ACS) recently recommended that CRC screening should start at the age of 45 in average-risk individuals independent of race and sex^1^ and the American College of Gastroenterology advises screening starting at the age of 45 in African Americans^2^. Three other American, as well as all European, including the Austrian, guidelines however, recommend starting screening for average-risk individuals at the age of 50 for both sexes^3–7^. However, just recently the USPSTF (U.S. Preventive Services task force) launched a new position paper that recommends starting colorectal screening at 45 years (class B recommendation)^8^.

The ACS justified the age decrease by epidemiologic data showing that there has been an increase in CRC incidence in subjects younger than 50 years in the last decades in the US^9^. A similar trend was observed in the European population: CRC incidences are rising in subjects younger than 50 years and declining in individuals over 50 years^10^.

Pearlman et al.^11^ showed that in early-onset CRC (CRC diagnosis below the age of 50), only 16% of affected patients had one pathogenic cancer susceptibility gene mutation. Therefore, heritability only explains a small proportion of early-onset CRC and a discussion about earlier screening appears prudent. Furthermore, younger patients are frequently diagnosed in advanced stages^12^ and have a lower 5-year survival rate^13^. Therefore, lowering the age recommendation for screening could improve outcomes. However, the change in age recommendations would redistribute resources to a younger population at lower absolute risk for CRC, as the incidence of adenoma, AA and CRC are rising with age^9,14,15^.

Furthermore, epidemiologic studies support the concept of significant sex and racial differences in CRC incidence. CRC and AA rates seem to be similar in both sexes until the age of 35, diverging thereafter with higher incidence in males, and a widening gap with increasing age^9^.

Pathophysiologic concepts for these age specific differences are incompletely understood. Established risk factors including genetics, molecular and histopathologic abnormalities as well as features of the metabolic syndrome, smoking, alcohol, dietary components (processed red meat and vegetable/fruit consumption), among others fail to identify those at higher risk. This suggests that sex itself might be an independent risk factor for CRC development^16,17^.

In 2011, Ferlitsch et al. analyzed an Austrian screening colonoscopy cohort and suggested that there should be different age recommendations based on sex, as numbers needed to screen for male patients at 45 to 49 were comparable to female patients between 55 and 59^18^.

However, a recent sex-specific and risk-factor adjusted analysis of European patients undergoing CRC screening is lacking. We, therefore, analyzed sex-specific, age and risk factor adjusted adenoma, AA and AN detection rates in a well-characterized Austrian CRC screening cohort.

## Methods

### Ethics statement

The study and data acquisition was performed according to the Helsinki Declaration and was approved by the local ethics committee (Ethics Commission for the Province of Salzburg, committee approval no. 415-E/1262/2–2010). Written informed consent was obtained from every participant and all assessments were performed according to national or international guidelines.

### Subjects

Participants were included from the Salzburg Colon Cancer Prevention Initiative, (SAKKOPI) a cohort consisting of 5943 patients (52% male and 48% female patients, median age 58.0 years, IQR 15.0) that were screened for colorectal cancer at a single Austrian center between July 2010 and June 2019. Patients from the general population were recruited after referral by their general practitioner or by self-assignment in an opportunistic screening program that was financially covered by health insurance at no cost for the patient.

The overall cohort consisted of 5943 patients (Supplementary Fig. S1). Of these, 154 were excluded as no colonoscopy was performed. As we wanted to address the question at which age screening should start, we excluded patients that were younger than 45 years and older than 60 years (2599 patients). In the next step, we further excluded patients with a family history of CRC in a first degree relative (335 patients) and 31 patients with an established diagnosis of inflammatory bowel disease. Thus, the final analysis comprised 2824 patients.

Patients were further divided into three age groups. Group 1 consisted of 521 patients between 45 and 49 years, group 2 of 1164 patients between 50 and 54 years and group 3 of 1139 patients between 55 and 60 years. Although the recommended screening age in Austria is 50, our cohort also included younger patients that were screened due to patient preference.

### Patient assessment

As previously reported in a smaller group of this cohort, patients participating were examined on two consecutive days^19,20^. Laboratory assessment as well as clinical examination was done on the first day, the colonoscopy exam on the second day. Patients completed a questionnaire about family and past medical history. Body mass index was calculated by dividing the weight by the squared body height. Overweight was defined as a body mass index (BMI) between 25.0 and 29.9 kg/m^2^ and obesity by a BMI ≥ 30.0 kg/m^2^.

Patients were categorized as being “never smokers”, “ever smokers” or “current smokers” based on their information about smoking habits. Patients were characterized as being alcohol abusers if they stated to drink ≥ 30 g (males) respectively ≥ 20 g (females) pure alcohol per day^21^. Dietary patterns were evaluated using a questionnaire. The amount of vegetable and fruit intake and red meat portions per week were quantified using an ordinal scale.

Patients were defined as being prediabetic if they had impaired fasting glucose (IFG) or impaired glucose tolerance (IGT). IFG was defined by a fasting glucose between 100 and 125 mg/dl and IGT was defined as a blood glucose of 140–199 mg/dl two hours after an oral glucose tolerance test (oGTT). Type 2 diabetes mellitus was present if a patient either had an HbA1c ≥ 6.5%, a fasting blood glucose ≥ 126 mg/dl, a blood glucose level of ≥ 200 mg/dl 2 h after the glucose tolerance test or took an antidiabetic drug^22,23^. Dysglycemia was present if patients had either diabetes or prediabetes. Arterial hypertension was defined as an office systolic blood pressure value ≥ 140 mmHg and/or an office diastolic blood pressure ≥ 90 mmHg or if the patient was on an antihypertensive drug^24^. Visceral obesity was defined if waist circumference was > 102 cm in men and > 88 cm in women^25^.

### Colonoscopy and histologic classification

Colonoscopy was performed according to recommendations by national and international guidelines and all performance measures were reached^26,27^.

All polyps were sent for histologic analysis and were characterized based on their macroscopic and histologic results. Polyps were classified as hyperplastic polyps, adenoma, AA, serrated lesions or carcinoma. An adenoma was defined as being advanced if (1) size was ≥ 1 cm, (2) high-grade dysplasia was present or (3) villous features were seen histologically^28,29^. An AN was present if a patient either was diagnosed with an AA or a carcinoma. Hyperplastic polyps ≥ 1 cm were not classified as advanced adenoma.

Polyps were furthermore classified by their location (proximal: if found in the cecum, ascending or transverse colon; distal: if found in the splenic flexure, the descending colon or sigmoid)^30^. Lesions in the rectum were counted separately.

### Quality measures for colonoscopy

Adenoma detection rate in the total cohort of 5789 patients (before excluding patients based on age, family history or past medical history) was 31.3% (38.2% for men and 23.8% for female), cecum intubation rate was 98.8% and the rate of adequate bowel preparation as defined by a Boston Bowel Preparation Score ≥ 6 was 98.5%^31^.

### Statistics

Continuous variables are expressed as mean (± standard deviation) and compared using T-test or analysis of variance (ANOVA). Categorical data are expressed as numbers or percentage. Chi-square test was applied to assess differences between proportion/distribution of categorical/ordinal characteristics between groups.

A propensity score for being female was calculated. The covariates for the propensity score included age (year), BMI (kg/m^2^) and LDL (mg/dl), triglycerides (mg/dl), as well as arterial hypertension, dysglycemia, smoking status, alcohol abuse, fruit/vegetable portions and meat meals per week as dummy variables. The propensity score was calculated using logistic regression. For the matched cohort, matching for propensity score using “nearest neighbor” matching was performed, the maximum allowed distance was 0.001. Males and females were matched 1:1.

Additionally, univariable and multivariable logistic regression models adjusting for the propensity score were built to evaluate unadjusted and adjusted gender differences for the primary and secondary outcome. The primary outcome was AA detection rate, and secondary outcomes were adenoma detection rate (ADR) and AN detection rate. Unadjusted odds ratios (OR), adjusted odds ratios (aOR) and respective 95% confidence intervals (CI) were obtained. Sensitivity analyses evaluating only patients in group 1, group 2 and group 3 were performed. The number needed to screen (NNS) were calculated by dividing the total number of subjects screened by the subjects with a pathologic finding in colonoscopy. All tests were two-sided, and a p-value of < 0.05 was considered statistically significant. SPSS version 23.0 (IBM, USA) and MedCalc Statistical Software version 19.1.3 (MedCalc Software bv Ostend, Belgium; https://www.medcalc.org; 2019) were used for all statistical analyses.

## Results

Comparing 1293 (46%) female to 1531 (54%) male patients (Table 1) mean age was similar in both groups (53.4 ± 4.1 years vs. 53.3 ± 4.2; p = 0.563). Significant differences were observed in almost all baseline characteristics. Women were less likely to be obese (39% vs. 51%; p < 0.001), had a lower rate of dysglycemia (38% vs. 54%; p < 0.001) and had a lower systolic and diastolic blood pressure (127.0 ± 16.7/79.4 ± 10.7 mmHg vs. 132.4 ± 17.6/81.9 ± 10.4 mmHg; p < 0.001). The intake of lipid-lowering drugs (both sexes 14%; p = 0.956) or aspirin (both sexes 17%; p = 0.729) were not different. We also analyzed intake of fruit or vegetables and found that female patients ate more portions of fruit/vegetables than male. Furthermore, men consumed significantly more meals with red meat per week than women (Table 1).

**Table 1:** Baseline characteristics of patients grouped by sex.

Patient characteristics	Female	Male	p-value
Number of patients (%)	1293 (45.8)	1531 (54.2)	
Age ~	53.4 (4.1)	53.3 (4.2)	0.563
**Metabolic characteristics**			
Body measurements			
Visceral obesity*	39.0	51.3	< 0.001
BMI kg/m^2^ ~	26.2 (5.3)	27.7 (4.2)	< 0.001
Waist circumference cm ~	90.0 (13.6)	100.0 (14.5)	< 0.001
Glucose metabolism			
Dysglycemia*	37.7	54.1	< 0.001
Diabetes*	12.7	18.7	< 0.001
Prediabetes*	25.0	35.4	< 0.001
HbA1c % ~	5.5 (0.5)	5.6 (0.7)	< 0.001
oGTT 2 h mg/dl ~	117.9 (31.7)	115.3 (37.2)	0.083
FBG mg/dl ~	96.4 (20.6)	103.6 (24.5)	< 0.001
Blood pressure			
Arterial hypertension*	53.1	61.9	< 0.001
SBP mmHg ~	127.0 (16.7)	132.4 (17.6)	< 0.001
DBP mmHg ~	79.4 (10.7)	81.9 (10.4)	< 0.001
Lipids			
Lipid lowering drugs*	13.8	13.8	0.956
Triglycerides mg/dl ~	109.5 (70.7)	146.5 (114.5)	< 0.001
LDL mg/dl ~	144.3 (39.1)	143.1 (38.9)	0.391
HDL mg/dl ~	65.4 (17.4)	51.7 (14.1)	< 0.001
**CRC risk factors**			
Addictive behaviours			
Ever smokers*	52.9	60.7	< 0.001
Active smokers*	31.3	31.3	1.000
Alcohol abusers^1^*	0.5	6.2	< 0.001
Medication			
ASS*	17.1	16.5	0.729
Nutrition			
> 5 portions fruit/vegetable per day*	21.7	6.5	< 0.001
3–4 portions fruit/vegetable per day*	23.8	15.8	< 0.001
1–2 portions fruit/vegetable per day*	41.2	49.8	< 0.001
< 1 portion fruit or vegetable per day*	13.3	27.9	< 0.001
> 4 red meat meals per week*	0.8	3.7	< 0.001
3–4 red meat meals per week*	11.5	23.0	< 0.001
< 3 red meat meals per week*	79.9	73.3	< 0.001

*BMI* body mass index; *oGTT* oral glucose tolerance test; *FBG* fasting blood glucose; *SBP* systolic blood pressure; *DBP* diastolic blood pressure; *LDL* low density lipoprotein; *HDL* high density lipoprotein; *ASS* aspirin.

*Numbers expressed as percentage; ~ numbers expressed as mean with standard deviation (in brackets).

^1^ > 30 g/day and > 20 g/day for males and females, respectively.

Age- and sex-specific findings of colonoscopy were evaluated (Table 2). The absolute rates of colorectal carcinoma were low and did not differ between age or sex groups.

**Table 2:** Sex and age specific colonoscopy findings.

	45–49			50–54			55–60			Total		
	Female	Male	p-value	Female	Male	p-value	Female	Male	p-value	Female	Male	p-value
Number of patients (%)	216 (16.7)	305 (19.9)		551 (42.6)	613 (40)		526 (40.7)	613 (40)		1293 (100)	1531 (100)	
Any adenoma*	17 (7.9)	70 (23.0)	p < 0.001	79 (14.3)	181 (29.5)	p < 0.001	119 (22.6)	215 (35.1)	p < 0.001	215 (16.6)	466 (30.4)	p < 0.001
Advanced adenoma*	5 (2.3)	16 (5.2)	p = 0.115	10 (1.8)	49 (8.0)	p < 0.001	23 (4.4)	48 (7.8)	p = 0.019	38 (2.9)	113 (7.4)	p < 0.001
Colorectal carcinoma*	2 (0.9)	2 (0.7)	p = 1.000	1 (0.2)	2 (0.3)	p = 1.000	3 (0.6)	2 (0.3)	p = 0.667	6 (0.5)	6 (0.4)	p = 0.780
Advanced neoplasia*	6 (2.8)	16 (5.2)	p = 0.191	11 (2.0)	49 (8.0)	p < 0.001	24 (4.6)	8.2	p = 0.016	41 (3.2)	115 (7.5)	p < 0.001

Crude event rates stratified for sex and age.

*Numbers expressed as total number of patients, percentage in brackets.

The prevalence of AA was lower in female patients (3% vs. 7%; OR 0.38 95% CI 0.26–0.55; p < 0.001) and remained so after propensity score adjustment (aOR 0.54 95% CI 0.34–0.86; p = 0.01). In age-adjusted sensitivity analysis, female patients had numerically lower numbers of AA than male patients in age group 1 and statistically significant lower numbers in age group 2 and 3 (age group 1: 2% vs. 5%; OR 0.43 95% CI 0.15–1.19; p = 0.10; age group 2: 2% vs. 8%; OR 0.21 95% CI 0.11–0.42; p < 0.001; age group 3: 4% vs. 8%; OR 0.54 95% CI 0.32–0.90; p = 0.019; Fig. 2). The NNS to detect an AA were similar in female age group 3 (NNS of 23) and male age group 1 (NNS of 19).

**Figure 1:**
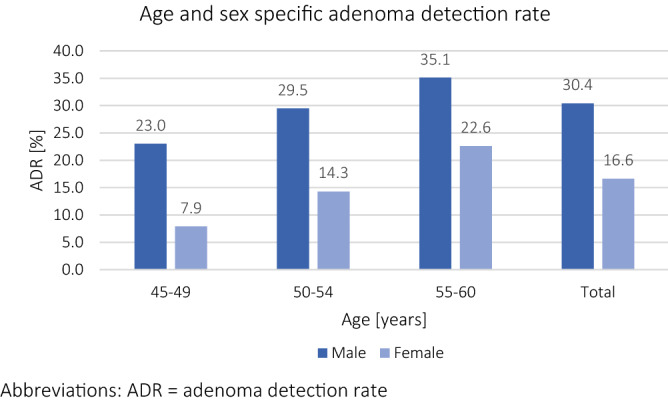
Age and sex specific adenoma detection rate. *ADR* adenoma detection rate.

The prevalence of any adenoma was also lower in females than in males (17% vs. 30%; OR 0.46 95% CI 0.38–0.55; p < 0.001) and remained so after propensity score adjustment (aOR 0.52 95% CI 0.41–0.66; p < 0.001). In both sexes, the rate of adenomas increased with age (Fig. 1). In age-group adjusted sensitivity analysis, in age group 1 (8% vs. 23%; OR 0.29 95% CI 0.16–0.50; p < 0.001), in age group 2 (14% vs. 30%; OR 0.40 95% CI 0.30–0.54; p < 0.001) and age group 3 (23% vs. 35%; OR 0.54 95% CI 0.42–0.70; p < 0.001) significantly fewer adenomas were found in females compared to males. Converted into NNS to detect an adenoma (Table 3), females showed a similar number (NNS of 4) in age group 3 compared to male patients in age group 1 (NNS of 4).

**Table 3:** Age and sex specific number needed to screen with colonoscopy to detect an adenoma, an advanced adenoma or an advanced neoplasia, respectively.

	45–49		50–54		55–60		Total	
	Female	Male	Female	Male	Female	Male	Female	Male
NNS to detect adenoma	12.7	4.3	7.0	3.4	4.4	2.9	6.0	3.3
NNS to detect advanced adenoma	44.1	19.2	55.7	12.5	22.9	12.7	34.5	13.5
NNS to detect advanced neoplasia	43.0	19.1	50.1	12.5	22.0	12.3	31.5	13.3

*NNS* number needed to screen.

**Figure 2:**
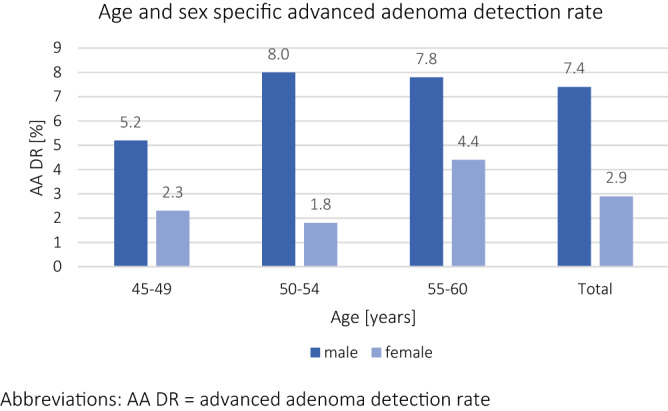
Age and sex specific advanced adenoma detection rate. *AA DR* advanced adenoma detection rate.

For AN, the prevalence was again lower in female patients (3% vs. 8%; OR 0.40 95% CI 0.28–0.58; p < 0.001) and remained so after propensity score adjustment (aOR 0.57 95% CI 0.36–0.90; p = 0.015). In age-adjusted sensitivity analysis, female patients had numerically lower numbers of AN than male patients in age group 1 and statistically significant lower numbers in age group 2 and 3 (age group 1: 3% vs. 5%; OR 0.52 95% CI 0.20–1.34; p = 0.18; age group 2: 2% vs. 8%; OR 0.23 95% CI 0.12–0.46; p < 0.001; age group 3: 5% vs. 8%; OR 0.54 95% CI 0.33–0.89 p = 0.015; Fig. 2). The NNS to detect an AA were therefore again similar in female age group 3 (NNS of 22) and male age group 1 (NNS of 19).

Locations of AA (proximal colon, distal colon or rectum) did not significantly differ between sexes (Supplement Table S1). Age-adjusted baseline characteristics of the total cohort are shown in Supplementary Table S2.

### Propensity-matched analysis

For further adjustment, a 1:1 propensity score matching of males and females (n = 557 for each group) was used to evenly distribute baseline risk. (Table 4). After matching differences in baseline characteristics and management were significantly reduced.

**Table 4:** Propensity matched analysis of female and male patients.

Patient characteristics	Female	Male	p-value
Number of patients	557	557	
Age ~	53.6 (4.1)	53.4 (4.0)	p = 0.297
**Metabolic characteristics**			
Body measurements			
Visceral obesity*	53.7	29.6	p < 0.001
BMI mean kg/m^2^ ~	26.9 (5.5)	26.8 (3.6)	p = 0.900
Waist circumference in cm ~	103.3 (11.2)	102.1 (7.3)	p = 0.034
Glucose metabolism			
Dysglycemia*	44.2	41.3	p = 0.364
Diabetes*	29.5	29.8	p = 0.948
Prediabetes*	14.7	11.5	p = 0.131
HbA1c % ~	5.6 (0.6)	5.5 (0.5)	p = 0.044
oGTT 2 h mg/dl ~	119.2 (33.6)	108.4 (29.5)	p < 0.001
FBG mg/dl ~	98.5 (22.9)	98.6 (16.8)	p = 0.942
Blood pressure			
Arterial hypertension*	61.2	59.4	p = 0.582
SBP mmHg ~	129.4 (17.9)	130.8 (15.2)	p = 0.139
DBP mmHg ~	80.4 (10.7)	81.0 (9.5)	p = 0.327
Lipids			
Lipid lowering drugs*	15.6	14.9	p = 0.803
Triglycerides mg/dl ~	120.0 (87.4)	121.0 (64.5)	p = 0.820
LDL mg/dl ~	145.0 (40.3)	146.3 (37.4)	p = 0.583
HDL mg/dl ~	64.6 (17.1)	53.8 (13.0)	p < 0.001
**CRC risk factors**			
Addictive behaviours			
Ever smokers*	54.2	53.0	p = 0.719
Active smokers*	31.9	28.4	p = 0.288
Alcohol abusers*	0.7	1.1	p = 0.753
Medication			
ASS*	17.1	17.8	p = 0.798
Nutrition			
> 5 portions fruit/vegetable per day*	10.6	11.1	p = 0.847
3–4 portions fruit/vegetable per day*	24.4	23.3	p = 0.725
1–2 portions fruit/vegetable per day*	49.0	47.8	p = 0.719
< 1 portion fruit or vegetable per day*	16.0	17.8	p = 0.472
> 4 red meat meals per week*	1.1	1.4	p = 0.789
3–4 red meat meals per week*	15.6	14.4	p = 0.615
< 3 red meat meals per week*	83.3	84.2	p = 0.745
Colonoscopy			
Any adenoma	18.1	29.1	**p < 0.001**
Advanced adenoma	3.8	7.4	**p = 0.012**
Advanced neoplasia	3.9	7.4	**p = 0.019**

*BMI* body mass index; *oGTT* oral glucose tolerance test; *FBG* fasting blood glucose; *SBP* systolic blood pressure; *DBP* diastolic blood pressure; *LDL* low density lipoprotein; *HDL* high density lipoprotein; *ASS* aspirin.

*Numbers expressed as percentage; ~ numbers expressed as mean with standard deviation (in brackets).

The prevalence of any kind of adenoma remained significantly lower in female patients compared to propensity score-matched males (any adenoma 18.1% vs. 29.1%; p < 0.001; any AA 4% vs. 7%; p = 0.012; any advanced adenoma 4% vs. 7%; p = 0.019).

## Discussion

In this study, evaluating a well-characterized Austrian CRC screening cohort of 2824 subjects, women had lower rates of colorectal adenoma, AA and AN compared to men. This sex-specific difference remained stable throughout all age groups. Additionally, a propensity-matched analysis correcting for metabolic and nutritional factors that significantly differed between sexes, evenly distributing these factors, confirmed that adenoma and advanced adenoma detection rates remained different between sexes independently of these factors.

Although there are various risk factors that promote colorectal adenoma and colorectal carcinoma development^32^, age and sex are dominant factors for incidence rates of colorectal diseases^14,15,33^.

Significant sex differences were also found in two big CRC screening trials, with 7% of women versus 11–13% of men between 50 and 65 having AA^15,33^. Also, differences in sensitivity and specificity of fecal occult blood tests were found—with men showing a higher sensitivity and positive predictive value compared to women^34^. This might be a reason for higher rates of missed cancers and differences in interval cancers, which are slightly elevated in women^35,36^ and should prompt a discussion about the optimal screening strategies including the sex of a patient. Although we could not find statistically significant differences in the location of advanced adenoma between sexes, other studies have shown that sex is related to disparities in the location of colorectal carcinoma. Women more often develop cancers in the proximal colon compared to men where CRC is more often located in the distal colon and rectum^37^. In a Norwegian study using flexible sigmoidoscopy, only covering the left-sided colon for screening, no mortality benefit for this screening approach for women was seen^38^.

Other reasons for these sex differences are under debate. Hormones might play a role, as nulliparous women have a higher risk of CRC, while women on hormone replacement therapy seem to have a lower risk^39^. Furthermore, microsatellite instability of colon cancers might be affected by estrogen^40^. Also, levels of hormones as parathyroid hormone (PTH) or vitamin D (VD) seem to have different effects in women and men^41,42^. Higher levels of PTH result in higher rates of distal colorectal adenoma and dysplasia in women but not in men^42^ and high levels of vitamin D were only protective for adenoma development in women but not in men^41^.

Further potentially contributing factors include different participation rates in screening examinations^38^, smoking rates, as well as a higher rate of metabolic syndrome in men^43^ and differences in lifestyle associated factors^32,44^.

Although there are various attempts to explain the differences between women and men, exact pathophysiologic mechanisms are still a matter of debate. Our propensity matched analysis, as well as other studies have shown that traditional risk factors alone cannot explain the differences between sexes^16^. Therefore, sex seems to be a particularly important risk factor for colorectal cancer development and this fact should prompt discussions about sex-specific recommendations in national and international CRC screening guidelines. Furthermore, it underlines that gender specified research is necessary to provide an optimal screening strategy for the individual person.

The second finding of this study was that in both sexes, the prevalence of adenoma and AA increased with age. Interestingly, in the youngest age group of male patients (45–49) analyzed already 5% were found to have an AA. As patients with a first degree relative with CRC were excluded, these patients are on average risk for colorectal carcinoma.

A rising incidence of CRC incidence in subjects younger than 50 years was seen in the last decades in the US and European populations^9,10^. This finding prompted the ACS to apply a microsimulation model to calculate CRC cases and deaths averted by screening as well as colonoscopies required to gain a life year^1^. This model estimated that the optimal cut-off for screening was 45 years, and the ACS lowered their screening recommendations in 2018 to 45 for both sexes^1^. Another model predicted, that lowering the starting age of screening to 45 means 160 to 784 additional lifetime colonoscopies, 22 to 27 life years gained, 2 to 3 fewer CRC cases and 1 averted death per 1000 people^45^. This benefit is opposed by 0.1 to 2 additional complications over the lifetimes of these 1000 individuals^45^. It was, however also shown, that risk of bleeding and perforation increased with age with the youngest population being at the lowest risk for complications^46,47^. These data, in our opinion, clearly depict that the favorable outcomes outweigh the harms of the procedure in young individuals and also led the USPSTF to change their recommendations in 2021^8^.

Another important, public health issue is the economic burden deriving from an extension of age recommendations. Here, a U.S. group questioned if the money could be invested more meaningfully by increasing the participation rate in older, higher risk adults where a lower number of colonoscopies would be sufficient to prevent CRC or colorectal cancer deaths^48^.

Overcoming the financial aspects, sex-specific recommendations could help in purposeful relocation of resources. In our analysis, we showed that NNS for adenoma, AA and AN rates were similar in male patients aged 45 to 49 years and female patients aged 55 and 60 years. A meta-analysis including 925.000 patients from America, Europe and Asia also showed that the relative risk for AA and colorectal carcinoma was found to be almost doubled for men within the same age classes^49^. Concordantly, women reached similar numbers needed to screen ten years later than men.

This 10-year incidence gap between sexes was also described in another large Austrian screening cohort^18^. Ferlitsch et al. showed that NNS for AA were similar in men between 45 and 49 and female between 55 and 59^18^. As patients with a positive family history for CRC were included as well as risk stratification based on metabolic risk factors was not feasible, our study adds valuable information, suggesting that this effect remained intact even after exclusion of patients suggestive of a genetic background and after propensity score adjustment for risk factors.

Limitations of our study clearly are that only Caucasian patients were included and that these data are therefore only applicable in this population group. Additionally, patients were referred to our center by their primary care physicians or by self-referral, which could prone the analysis to selection bias, which might be especially true for the youngest age group, as no general recommendation for this age exists. However, every analysis using data from non-invasive and invasive procedures requires patient consent and necessarily misses the patients who either do not seek medical help or do not consent to colonoscopy. Thus, this limitation is applicable to all studies reporting endoscopy results. Also, data on dietary habits as fiber or dairy intake, exact amounts of sugar-sweetened beverages or physical activity were only available in a limited number of subjects and therefore not included in the analyses. Furthermore, absolute rates of CRC were low in this study, therefore this analysis was underpowered to detect differences between age and sex groups. However, Song et al. showed recently in a large matched cohort study that the presence of a tubular adenoma, a tubulo-villous or a villous adenoma at index colonoscopy was associated with an increased risk for colorectal carcinoma over lifetime, validating the use of adenoma and advanced adenoma as proxy variables for future CRC^50^. Another limitation is the lack of an analysis of the prevalence of serrated lesions. Serrated lesions were just recently included in the classification system of polyps and represent a diagnostic challenge with a high interobserver variability^51^. Recently, a systematic review estimated the global prevalence to range between 2.6% and 10.5% with a pooled prevalence of 4.6%^52^. However, the prevalence varied significantly among the literature over time, reporting a prevalence of sessile serrated lesions of 1.6% in 2010 to 20.1% in 2017, highlighting the increased awareness for this entity during the last two decades^53,54^. Of note, the prevalence of sessile serrated lesions was 0.9% between 2010 and 2015 and 3.3% between 2016 and 2019 in our study population. However, this increased global awareness and knowledge about serrated lesions makes a comparison over the time period in our study population difficult and therefore serrated lesions were not included in the analysis.

**Figure 3:**
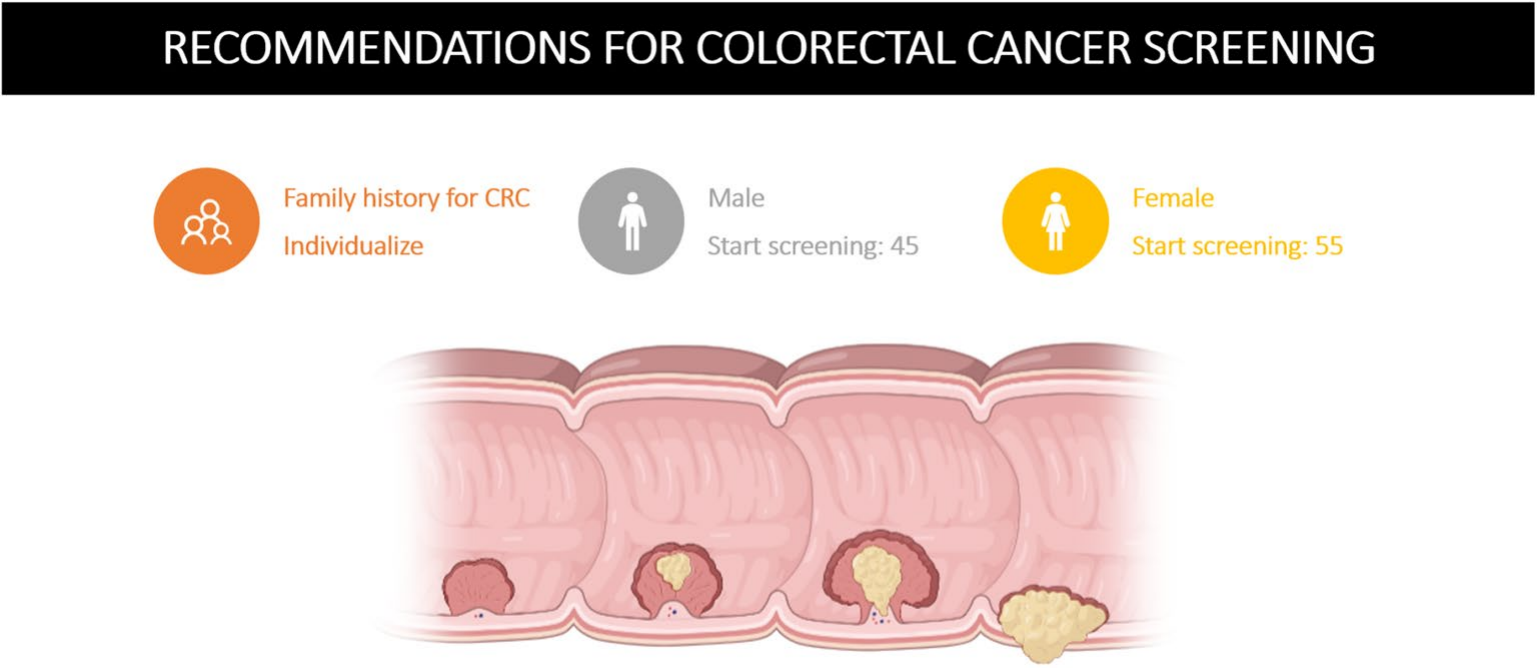
Age, sex and risk adjusted screening algorithm recommendation. *CRC* colorectal cancer; Created with BioRender.com.

In conclusion, this study represents contemporary real-world screening data of a well-characterized cohort of Caucasian subjects. Rates of adenoma, AA and AN were significantly lower in female patients. This finding remained after propensity score adjustment for numerous risk factors of CRC and in all age-adjusted sensitivity analyses. The NNS in males aged 45–49 years was comparable to the NNS of females aged 55–60 years. Therefore, this study adds information to the discussion about sex differences and age recommendations for colorectal cancer screening in Caucasian individuals, enhancing a discussion about a reduction in screening age for males to 45 years and an increase to 55 years in females thereby potentially redistributing resources to a high-risk population. Here, further research on the optimal technique of screening is also pending. A graphical illustration of this proposed new screening strategy based on age and sex is summarized in Fig. 3. Nevertheless, as this is only a retrospective analysis of a cross sectional study, randomized controlled trials evaluating this finding in a bigger cohort, including different ethnicities focusing on sex differences, should follow to support our findings.

## Conclusion

In this study evaluating an Austrian CRC screening population, adenoma, AA and AN detection rates were consistently lower in female than in male patients. This finding persisted after propensity score adjustment and in all age-adjusted sensitivity analyses, supporting evidence that sex alone is one of the most important risk factors for CRC. The NNS in males aged 45–49 years was numerically comparable to the NNS of females aged 55–60 years. This should lead to discussions about the optimal first screening age based on individual risk factors and sex.

## Supplementary Information

Supplementary Information.
