# Supplementary material for: A sex-specific propensity-adjusted analysis of colonic adenoma detection rates in a screening cohort
Source: Sci Rep. 2021 Sep 7;11:17785. doi: 10.1038/s41598-021-97163-0 (PMC8423798; doi:10.1038/s41598-021-97163-0)
Supplement: Supplementary file 1 — Supplementary Information. [file 41598_2021_97163_MOESM1_ESM.docx]

**SUPPLEMENT**

**TITLE PAGE**

**Full text**

A sex-specific propensity-adjusted analysis of colonic adenoma detection rates in a screening cohort

**Author List and Affiliations**

Sarah Wernly^1^, Bernhard Wernly^2,3^, Georg Semmler^1,4^, Sebastian Bachmayer^1^, David Niederseer^5^, Felix Stickel^6^, Ursula Huber-Schönauer^1^, Elmar Aigner^7^, Christian Datz^1^

^1^ Department of Internal Medicine, General Hospital Oberndorf, Teaching Hospital of the Paracelsus Medical University Salzburg, Oberndorf, Salzburg, Austria

^2^ Clinic of Internal Medicine II, Department of Cardiology, Paracelsus Medical University of Salzburg, Salzburg, Austria

^3^ Department of Medicine, Karolinska Institutet, Karolinska University Hospital, Stockholm, Sweden

^4^ Department of Internal Medicine III, Division of Gastroenterology and Hepatology, Medical University of Vienna, Vienna, Austria

^5^Department of Cardiology, University Hospital Zurich, Zurich, Switzerland

^6^ Department of Gastroenterology and Hepatology, University Hospital of Zurich, Zürich, Switzerland

^7^ First Department of Medicine, Paracelsus Medical University Salzburg, Salzburg, Austria

**Corresponding Author**

Christian Datz, M.D.

Department of Internal Medicine,

General Hospital Oberndorf, Teaching Hospital of the Paracelsus Medical University Salzburg

Paracelsusstraße 37

5110 Oberndorf, Austria

Phone: +43 6272 43340

E-Mail: [c.datz@kh-oberndorf.at](mailto:c.datz@kh-oberndorf.at)

**Supplementary Figure. Patient flow chart**


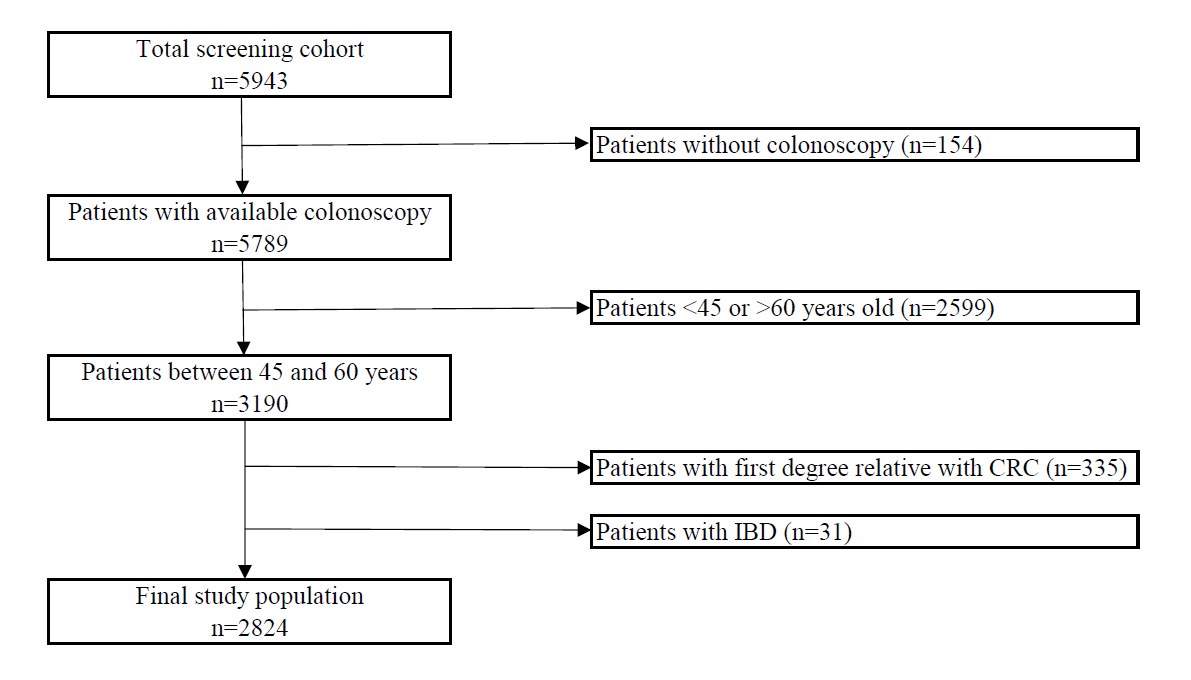


**Supplement Table 1. Location of advanced adenoma in male and female patients**

|  | **Female** | **Male** | **p-Value** |
| --- | --- | --- | --- |
| Proximal colon * | 43.6 | 38.9 | p=1.000 |
| Distal colon * | 38.5 | 42.1 | p=0.457 |
| Rectum * | 17.9 | 19.0 | p=0.819 |

*numbers expressed as percentage of all advanced adenoma in the specific sex group

**Supplement table 2. Baseline characteristics of patients grouped by age**

| **Patient characteristics** | | | **45-49** | **50-54** | **55-60** | **Total** | **p-value** |
| --- | --- | --- | --- | --- | --- | --- | --- |
|  |  | Number of patients (%) | 521 (18.4) | 1164 (41.2) | 1139 (40.3) | 2824 (100) |  |
|  |  | Female * | 41.5 | 47.3 | 46.2 | 45.8 | 0.077 |
| **Metabolic characteristics** | Body measurements | Visceral obesity * | 38.4 | 41.7 | 50.4 | 44.6 | <0.001 |
|  |  | BMI kg/m² ~ | 26.7 (4.6) | 26.8 (4.7) | 27.4 (4.8) | 27.0 (4.8) | 0.001 |
|  |  | Waist circumference cm ~ | 94.4 (15.0) | 94.6 (15.2) | 96.8 (14.6) | 95.5 (15.0) | 0.001 |
|  | Glucose metabolism | Dysglycemia * | 41.3 | 43.0 | 52.6 | 46.5 | <0.001 |
|  |  | Diabetes * | 13.4 | 14.9 | 18.1 | 15.9 | 0.026 |
|  |  | Prediabetes * | 27.9 | 28.0 | 34.6 | 30.6 | 0.001 |
|  |  | HbA1c % ~ | 5.4 (0.5) | 5.5 (0.6) | 5.7 (0.7) | 5.6 (0.6) | <0.001 |
|  |  | oGTT 2h mg/dl ~ | 111.5 (30.5) | 115.1 (32.5) | 120.4 (38.3) | 116.4 (43.7) | <0.001 |
|  |  | FBG mg/dl ~ | 97.0 (14.4) | 98.9 (22.9) | 103.3 (26.0) | 100.3 (23.1) | <0.001 |
|  | Blood pressure | Arterial hypertension * | 53.2 | 56.5 | 61.3 | 57.8 | 0.004 |
|  |  | SBP mmHg ~ | 125.5 (17.1) | 129.0 (17.7) | 132.9 (18.8) | 129.9 (18.3) | <0.001 |
|  |  | DBP mmHg ~ | 79.3 (10.8) | 80.8 (10.4) | 81.4 (10.7) | 80.8 (10.6) | 0.001 |
|  | Lipids | Lipid lowering drugs * | 14.0 | 14.2 | 13.3 | 13.8 | 0.837 |
|  |  | Triglycerides mg/dl ~ | 128.3 (96.0) | 126.7 (104.5) | 132.6 (93.6) | 129.5 (98.7) | 0.369 |
|  |  | LDL mg/dl ~ | 139.3 (38.8) | 144.3 (39.4) | 145.0 (39.4) | 143.6 (39.0) | 0.015 |
|  |  | HDL mg/dl ~ | 56.1 (16.3) | 58.9 (18.6) | 57.9 (15.7) | 57.9 (17.1) | 0.008 |
| **CRC risk factors** | Addictive behaviours | Ever smokers * | 57.2 | 58.2 | 56.0 | 57.1 | 0.641 |
|  |  | Active smokers * | 35.0 | 32.7 | 28.2 | 31.3 | 0.056 |
|  |  | Alcohol abusers * | 3.6 | 3.7 | 3.5 | 3.6 | 0.966 |
|  | Medication | ASS * | 16.6 | 16.9 | 16.7 | 16.8 | 0.984 |
|  | Nutrition | > 5 portions fruit/vegetable per day * | 11.2 | 14.6 | 13.4 | 13.5 | 0.237 |
|  |  | 3-4 portions fruit/vegetable per day * | 16.7 | 20.3 | 20.0 | 19.5 | 0.275 |
|  |  | 1-2 portions fruit/vegetable per day * | 46.1 | 42.3 | 49.3 | 45.8 | 0.010 |
|  |  | < 1 portion fruit or vegetable per day * | 26.0 | 22.8 | 17.4 | 21.2 | <0.001 |
|  |  | > 4 red meat meals per week * | 2.3 | 2.3 | 2.5 | 2.4 | 0.936 |
|  |  | 3-4 red meat meals per week * | 18.4 | 16.8 | 18.8 | 17.9 | 0.546 |
|  |  | < 3 red meat meals per week * | 79.3 | 80.9 | 78.7 | 79.7 | 0.512 |

Abbreviations: BMI = body mass index; oGTT = oral glucose tolerance test; FBG = fasting blood glucose; SBP = systolic blood pressure; DBP = diastolic blood pressure; LDL = low density lipoprotein; HDL = high density lipoprotein; ASS = aspirin; * numbers expressed as percentage; ~ numbers expressed as mean with standard deviation (in brackets)
